# Liver Fibrosis Indices Predict the Severity of SARS-CoV-2 Infection

**DOI:** 10.3390/jcm11185369

**Published:** 2022-09-13

**Authors:** Lucilla Crudele, Fabio Novielli, Stefano Petruzzelli, Stefano Battaglia, Antonio Francesco Maria Giuliano, Rosa Melodia, Chiara Maria Morano, Paola Dell’Aquila, Renata Moretti, Luigi Castorani, Roberto Salvia, Gianfranco Inglese, Nicola Susca, Lucrezia dell’Olio, Francesca Falcone, Mariapaola Castaldo, Carlo De Matteis, Carlo Sabbà, Antonio Moschetta

**Affiliations:** 1 Department of Interdisciplinary Medicine, University of Bari “Aldo Moro”, Piazza Giulio Cesare 11, 70124 Bari, Italy; 2 Medicina e Chirurgia d’Accettazione E d’Urgenza, Azienda Ospedaliero-Universitaria Policlinico di Bari, 70124 Bari, Italy; 3 Medicina Sub-Intensiva, Presidio Maxi-Emergenze Fiera del Levante, Azienda Ospedaliero-Universitaria Policlinico di Bari, 70124 Bari, Italy; 4 Medicina Interna Universitaria Cesare Frugoni, Azienda Ospedaliero-Universitaria Policlinico di Bari, 70124 Bari, Italy

**Keywords:** COVID-19, liver fibrosis, non-alcoholic steatohepatitis, non-alcoholic fatty liver disease, non-invasive liver fibrosis scores

## Abstract

Discovering novel risk and prognostic factors for COVID-19 may help not only in reducing severity and mortality but also in creating targeted therapies considering patients’ individual features. Liver fibrosis is considered a complication in Non-alcoholic Fatty Liver Disease (NAFLD), it is a feature of steatohepatitis (NASH), and it has already been related to an increased risk for a wide range of diseases. Here, we aimed to define if any parameter assessing metabolic status has predictive power in identifying inpatients at risk for poorer prognosis and an increased mortality from COVID-19. This retrospective study was conducted at the Sub-Intensive Medicine Care Unit of the Presidio Maxi-Emergenze Fiera del Levante, Azienda Ospedaliero-Universitaria Policlinico di Bari, Italy. We evaluated 271 inpatients with moderate-to-severe SARS-CoV-2-related respiratory failure by comparing biochemical features and non-invasive liver fibrosis scores among discharged, transferred to Intensive Care Units (ICU) and non-survivor patients. Moreover, by performing ROC curves, we defined cut-off values to predict mortality and disease severity for each score. We found that non-invasive scores of liver fibrosis, obtained at day of admission, such as AAR (*p* < 0.001), FIB-4 and mFIB-4, FORNS, and AARPRI (*p* < 0.05) strongly predict not only in-hospital mortality but also the length of hospitalization and eventual admission to ICU. FIB-4 was the best score to identify non-survivor patients (sensitivity of 80% and specificity of 63%) and predict the need for ICU or mortality (71% of sensitivity and 65% of specificity), with a cut-off value of 1.94. Therefore, we present the predictive power and the cut-off values of several liver fibrosis scores here for disease severity and mortality in SARS-CoV-2 in-patients and we proposed the use of the present scores to identify ab initio the clinical therapeutic and diagnostic protocols for high-risk patients.

## 1. Introduction

SARS-CoV-2 enters the peripheral blood from the lungs and spreads into cells expressing angiotensin-converting enzyme 2 (ACE-2), which represents the cognate receptor of the envelope Spike protein of SARS-CoV-2. Then, the intracellular lifecycle of the virus starts. The infected host cells trigger an immune response with the recruitment of T lymphocytes, monocytes, and neutrophils [1]. In severe COVID-19, the immune system overactivation results in a “cytokine storm” characterised by the release of high levels of some cytokines into the circulation, especially Interleukin-6 (IL-6) and Tumour Necrosis Factor- α (TNF-α), causing a local and systemic inflammatory response [2]. In addition to IL-6 and TNF-α, the binding of SARS-CoV-2 to the Toll-Like Receptor (TLR) leads to the release of Interleukine-1β (IL-1β), which mediates lung inflammation that can evolve into fibrosis, responsible for the well-known severe pulmonary manifestations [3].

Similarly, the inflammation and release of a plethora of cytokines and adipocyte-specific hormones are linked to excessive fat accumulation, specifically visceral adiposopathy, representing a risk factor for cardiovascular, metabolic and chronic obesity-related diseases, including cancer [4].

On the one hand, patients with increased abdominal obesity show a decreased diaphragmatic excursion that compromises the pulmonary function in a supine position, making ventilation more difficult [5]. Moreover, the impact of obesity on respiratory diseases is complex and reaches beyond the obvious physical and mechanical effects of weight gain and its associated metabolic and inflammatory disorders. Elevated levels of IL-6, Interleukin-8 (IL-8), TNF-α, C-Reactive Protein (CRP), leptin, and a lower level of adiponectin may represent the pathogenetic link between obesity-induced hypoxemia and respiratory disorders [6]. Furthermore, obesity is strongly linked with respiratory symptoms and diseases, including exertional dyspnea, obstructive sleep apnea syndrome (OSAS), obesity hypoventilation syndrome (OHS), chronic obstructive pulmonary disease (COPD), asthma, pulmonary embolism, and aspiration pneumonia [7]. On the other hand, obesity and its related conditions, especially type 2 diabetes and hypertriglyceridemia, are major contributors to the current epidemic of Non-Alcoholic Fatty Liver Disease (NAFLD) [8]. Non-Alcoholic Steatohepatitis (NASH) is the accumulation of fat in hepatocytes which causes inflammation, cell death, and fibrous scarring resulting in the disruption of the normal hepatic architecture and hepatic dysfunction. Since chronic liver injury results in repeated tissue damage that leads to an imbalance between extracellular matrix production and dissolution, steatohepatitis is associated with liver fibrosis. Fibrosis does not cause symptoms but can lead to portal hypertension, in which scar tissue alters hepatic portal flow, and cirrhosis. Currently, metabolic causes associated with chronic insult leading to liver fibrosis are much more common. Hepatic fibrosis is the most important factor of mortality in NAFLD, since the risk of death from hepatitis increases exponentially with an increasing level of fibrosis [9]. Although liver biopsy appears to be the gold standard for staging liver fibrosis, alternative methods of evaluation are increasingly validated and used, especially because they are non-invasive, clinical, and cheap, including several variables, such as age, anthropometric data, and laboratory values. Consequently, non-invasive clinical assessment systems have gained validity as first-line tools in patients with hepatic fibrosis. Moreover, in NAFLD patients, such non-invasive scoring systems are good predictors of morbidity and mortality and were found to have an additive value in predicting the development of hepatic and extra-hepatic cancers [10].

AAR, the ratio of Aspartate Transaminase (AST) to Alanine Transaminase (ALT), which is typically less than 1, can rise to greater values as fibrosis and cirrhosis develop. According to a study conducted by Giboney et al., 87 percent of patients with an AAR of 1.3 or less had NASH (87 percent sensitivity, 84 percent specificity). The severity of NASH as measured by the degree of fibrosis increased, as did the AAR. The mean ratio of 1.4 was found in patients with cirrhosis related to NASH. Wilson’s disease can cause the AAR to exceed 4. In short, certain AARs are suggestive of certain conditions. Consequently, since there is significant overlap between AAR in different conditions, and the exact mechanism of AAR alteration in the progression of liver disease is unclear, its accuracy in predicting the degree of fibrosis and the presence of cirrhosis is controversial and this ratio cannot be used alone to make a diagnosis [11].

Fibrosis-4 index (FIB-4) is a non-invasive score used to assess liver fibrosis in outpatient settings. The index is considered to be accurate, non-invasive, and easily available, and may be useful in evaluating patients with Hepatitis-C Virus (HCV), NAFLD, and other liver complications [12], although a high false positive rate has been detected for advanced fibrosis in older patients [13].

The modified fibrosis-4 (mFIB-4) index was elaborated on as an instrument to assess the stage of liver fibrosis in patients with chronic hepatitis B (CHB) or C (CHC). However, it is used for the detection of advanced liver fibrosis in all patients with chronic liver disease [14].

The FORNS index is based on the assessment of four routine parameters (age, platelets count, cholesterol, and gamma-glutamyl transferase (GGT)). The most important study to evaluate its diagnostic accuracy considered a cohort of 250 patients with CHC. The FORNS index is able to exclude the presence of severe fibrosis with a Negative Predictive Value of 96% and to identify the presence of severe fibrosis with a Positive Predictive Value of only 66%. It is therefore a useful test in identifying patients with minimal fibrosis but it has limited value in identifying patients with more advanced liver disease [15].

APRI score (AST to Platelet Ratio Index) is a non-invasive index for the assessment of liver fibrosis in patients with viral hepatitis, and represents an alternative to liver biopsy in the follow-up of patients with hepatitis C and liver cirrhosis in an outpatient setting. In a meta-analysis of 40 studies, it was concluded that an APRI score of above 1.0 presents a sensitivity of 76% and a specificity of 72% for predicting cirrhosis. An APRI score of above 0.7 presents a sensitivity of 77% and a specificity of 72% for predicting significant liver fibrosis [16]. It is likely that APRI alone is not sensitive enough to rule out significant diseases. Some evidence suggests that the use of multiple indices in combination (such as APRI plus FibroTest) or an algorithmic approach could result in greater diagnostic accuracy than using APRI alone [17].

AARPRI (AAR to Platelet Ratio Index) represents another important non-invasive score for the evaluation of hepatic fibrosis. In particular, it can be considered as one of the most reliable scores in the diagnosis of advanced stages of liver fibrosis. Moreover, AARPRI was also proposed as a predictor for chronic liver disease-associated complications [18,19]. All formulas used for calculating non-invasive liver fibrosis scores are summarised in Table 1.

The aim of this study was to identify novel metabolic parameters such as liver fibrosis scores at admission with the putative ability to predict disease severity and mortality in COVID-19 patients.

## 2. Materials and Methods

### 2.1. Study Design

The initial phase of the study focused on analysing the bio-humoral and haemato-chemical parameters and liver fibrosis scores to determine metabolic abnormalities in the study population. In a second phase, attention was focused on identifying whether there were statistically significant differences between patients who died during hospitalization and patients discharged home or moved to Intensive Care Unit (ICU). We analysed and correlated the various markers, identifying the most representative ones in predicting mortality and prognosis, also studying which of these parameters showed a correlation with the length of hospitalization in our Unit.

### 2.2. Study Participants

Patients’ recruitment, and clinical and biochemical analyses were registered consecutively in the electronic health register of the Medicina Sub-Intensiva Unit of Presidio Maxi-Emergenze (MSI-PME) at Teaching Hospital Policlinico di Bari, Italy, from April 2021 to April 2022. Admitted patients presented respiratory failure due to SARS-CoV-2 infection and required oxygen therapy up to noninvasive ventilation. A total of 443 patients with 18 or more years were initially enrolled in this study, among which 148 were excluded because biochemical data such as AST, ALT, GGT, or blood count were lacking on day 1, since the data were recorded before the admission to our ward, at the Emergency Department. Patients with previous viral hepatitis, cirrhosis, benign liver tumours (BLT), primary liver cancer (PLC) or hepatic metastasis at baseline (*n* = 13) and who admitted POTUS (*n* = 11) were excluded from the study. Acute heart failure and other acute diseases were excluded before admission to our unit.

In the end, statistical analysis was performed on a total population of 271 patients (149 males, 122 females). 

### 2.3. Baseline Evaluation and Biochemical Measurements

All participants underwent a detailed anamnesis and physical examination at admission. Unfortunately, precise anthropometric assessment at time 0 was not performed for all patients due to their critically ill status. Morning blood samples were obtained after 12 h of fasting from the antecubital veins of patients on the first day after admission to our unit. After blood clotting and centrifugation, serum was processed for an analysis of the biochemical markers of glucose and lipid metabolism. Liver, renal, thyroid and inflammatory markers were also studied following standardised biochemical procedures. All biochemical measurements were centralised and performed in the ISO 9001 certified laboratories of the University Hospital of Bari. Specifically, a complete blood count with a determination of the leukocyte subpopulation was performed. Measurements of total and High-Density Lipoprotein cholesterol (HDL-c), fasting plasma glucose (FPG), and triglycerides (TG) were obtained using an enzymatic colorimetric assay (Siemens, Erlangen, Germany). CRP was performed via nephelometry (Siemens, Erlangen, Germany). The low-density lipoprotein cholesterol (LDL-c) level was obtained using the Friedewald formula; the Neutrophil to Lymphocyte Ratio (NLR) and Monocyte to HDL-c ratio (MHR) were calculated manually.

### 2.4. Statistical Analysis

Descriptive statistical analyses of the study sample were performed, and their results are expressed as mean ± standard error of the mean (SEM) and frequencies (%), depending on the nature of variables. Comparisons of socio-demographic and clinical variables between two groups were conducted with the *t*-test (for continuous variables) and the Pearson χ^2^ test (for categorical variables). Analyses between more than two groups were performed through one-way analysis of variance (ANOVA), which was followed, where required, by Bonferroni’s post hoc test. The correlation between continuous variables was also analysed and estimated using Pearson’s Correlation Coefficient (r).

The receiver–operating characteristic (ROC) curves were used to determine the optimum cut-off levels of non-invasive liver scores in predicting mortality and severity of SARS-CoV-2 infection. Empirical ROC curves were plotted for these variables along with a calculation of the area under the curve (AUC) with 95% confidence intervals and one-sided upper p-values for the null hypothesis AUC = 0.5. The condition variable for severity was in-hospital death or ICU admission.

*p*-values lower than 0.05 were considered significant. All analyses were performed using the NCSS 12 Statistical Software, version 12.0.2018 (NCSS, LLC Company, Kaysville, UT, USA) and GraphPad Prism, version 9.1.0 (GraphPad Software; San Diego, CA, USA).

## 3. Results

### 3.1. Baseline Characterization of the Study Population

The average age of our 271 patients was 70. Mean length of the stay in our Unit was 13 days. A total of 38 patients (14%, 19 males and 19 females) died during hospitalization in our unit, while 233 (130 males and 103 females) were still alive when they moved from our unit. Of them, 208 patients (76.8%, 117 males and 91 females) were discharged, while 25 patients (9.2%, 3 males and 12 females) were transferred to ICU due to the worsening of their clinical conditions.

As shown in Table 2, non-survivor patients were significantly older (*p* < 0.001) and exhibited increased lymphocytopenia (*p* < 0.05) and glycemia (*p* < 0.001) at admission. Surprisingly, no significant differences were found for inflammation markers (White Blood Cells, CRP, Erythrocytes Sedimentation Rate, Ferritin) except for Procalcitonin (*p* < 0.05). With regard to lipid assessment, total cholesterol and LDL-cholesterol (LDL-c) were significantly lower in the non-survivor group (*p* < 0.05), while no statistical differences were found for AST, GGT, Total Bilirubin, and NT-proBNP. Myocitolysis parameters such as lactate dehydrogenase (LDH) (*p* < 0.001), creatine phosphokinase (CPK) (*p* = NS), myoglobin (*p* < 0.05), and troponin (*p* < 0.05) were all increased in the second group.

**Figure 1:**
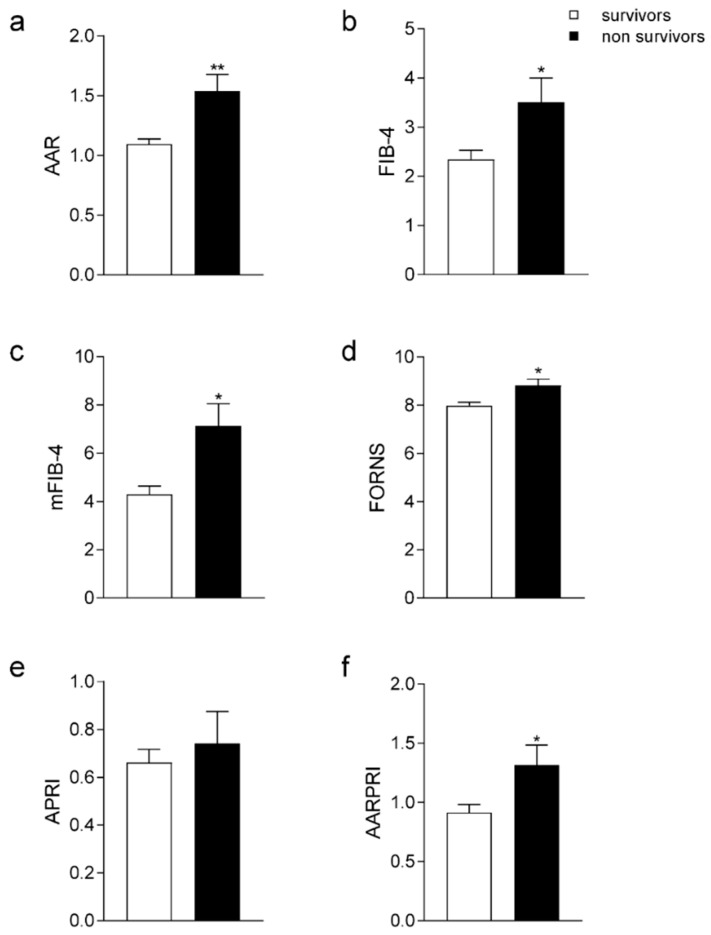
Comparison of Liver Fibrosis Scores in survivor and non survivor patients. Comparison of AAR (**a**), FIB-4 (**b**), mFIB-4 (**c**), FORNS (**d**), APRI (**e**), AARPRI (**f**) scores between survivors and non survivors. Data are presented as mean ± SEM. Statistical significance was assessed by Student *t*-test (* *p* < 0.05, ** *p* < 0.001). Abbreviations: AST to ALT ratio: AAR; fibrosis-4 index: FIB-4; modified FIB-4: mFIB-4; AST to Platelet Ratio Index: APRI; AAR to Platelet ratio: AARPRI.

Considering the liver fibrosis non-invasive indices, non-survivor patients presented significantly increased AAR (*p* < 0.001), FIB-4, (*p* < 0.05), mFIB-4 (*p* < 0.05), FORNS (*p* < 0.05), and AARPRI (*p* < 0.05) scores, but no significant difference was observed for APRI (Figure 1).

### 3.2. Liver Fibrosis Scores among Discharged, Admitted to Intensive Care Unit (ICU), and Non-Survivor Patients

To better understand if these scores might predict not only the mortality but also the evolution of critical disease and prognosis, we then performed a one-way ANOVA test by dividing our population into three groups according to their final outcomes, i.e., if they were discharged after clinical resolution, moved to ICU because of the worsening of their conditions, or died during hospitalization. The comparison was significantly different for AAR (*p* < 0.001), FIB-4 (*p* < 0.05), mFIB-4 (*p* < 0.05), FORNS (*p* < 0.01), and AARPRI (*p* < 0.05), but not for APRI.

**Figure 2:**
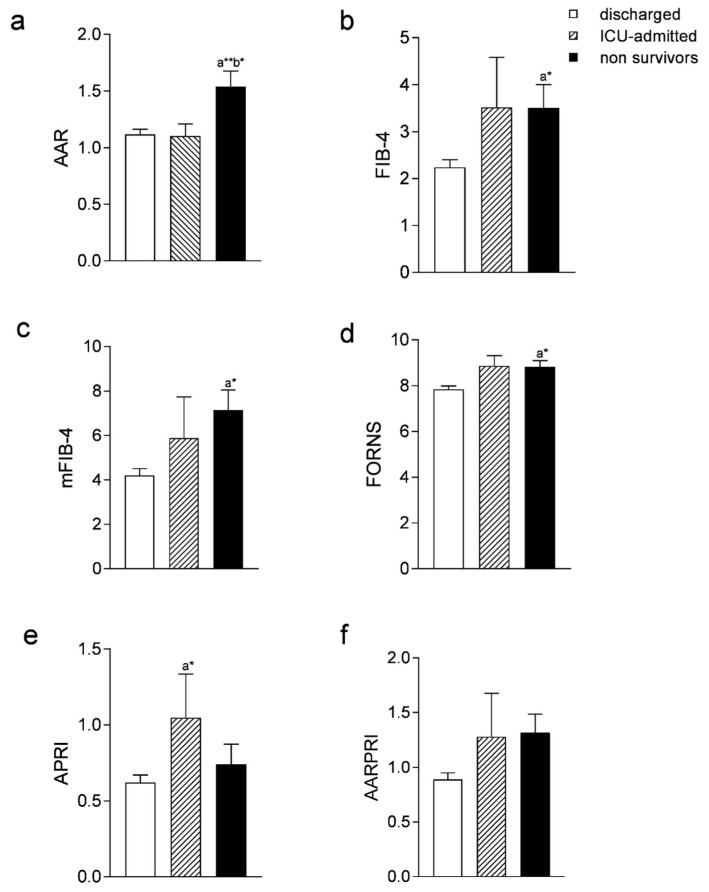
Comparison of Liver Fibrosis Scores among discharged, admitted to Intensive Care Unit (ICU), and non-survivor patients. Comparison of AAR (**a**), FIB-4 (**b**), mFIB-4 (**c**), FORNS (**d**), APRI (**e**), AARPRI (**f**) scores among discharged, ICU-admitted, and non survivor patients. Data are presented as mean ± SEM. Comparisons were performed using one-way ANOVA test followed by Bonferroni’s post hoc test. Multiple comparison was performed by Student *t*-test. Lowercase letter in the subfigures indicates significant difference (* *p* < 0.05, ** *p* < 0.001) between two groups: (**a**) for discharged patients, (**b**) for ICU admitted patients. Abbreviations: AST to ALT ratio: AAR; fibrosis-4 index: FIB-4; modified FIB-4: mFIB-4; AST to Platelet Ratio Index: APRI; AAR to Platelet ratio: AARPRI.

APRI only showed a statistically significant difference when compared by T-student test between ICU-admitted and survivor patients (Figure 2e, *p* < 0.05). Multiple comparisons also showed that only AAR (Figure 2a) was significantly higher in non-survivors versus either discharged (*p* < 0.001) or ICU-admitted (*p* < 0.05) patients, whereas other comparisons did not reveal any difference between these two groups.

**Figure 3:**
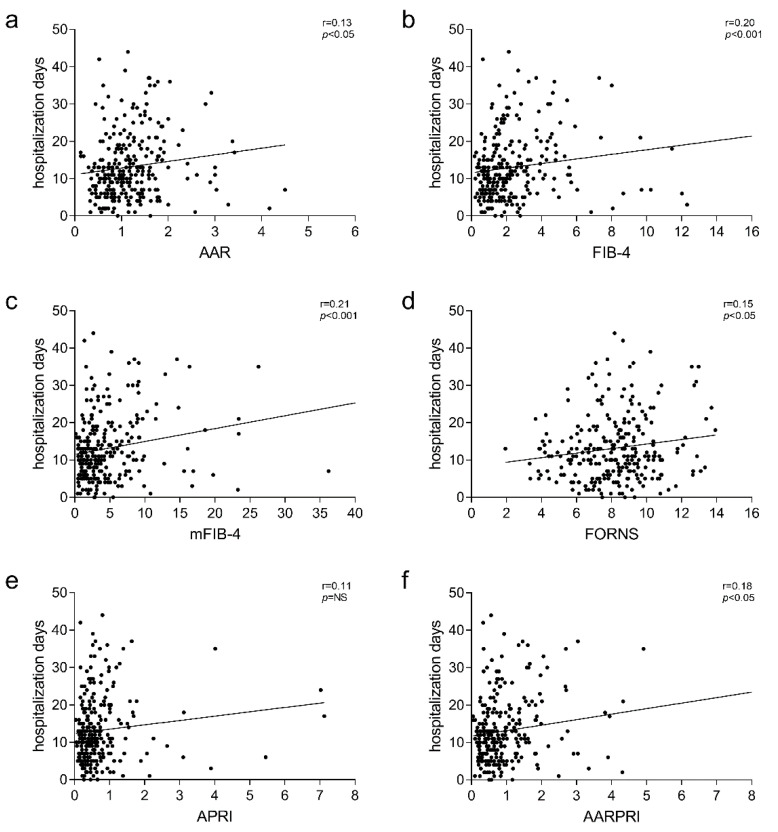
Correlation analysis of AAR (**a**), FIB-4 (**b**), mFIB-4 (**c**), FORNS (**d**), APRI (**e**), AARPRI (**f**) with hospitalization days in the whole population. The correlation was analysed and estimated using Pearson’s Correlation Coefficient (r). (*p*) indicates statistical significance. Each black dot represents a single patient. Abbreviations: AST to ALT ratio, AAR; fibrosis 4 index, FIB-4; modified FIB-4, mFIB-4; AAR to Platelet ratio, AARPRI.

Thus, assuming that the prognosis correlates with the length of the stay, we tried to deepen the correlation between Liver Fibrosis Indices and the days of hospitalization in MSI-PME, finding that all of them showed significant correlations (Figure 3a–d,f, *p* < 0.05), apart from APRI (Figure 3e).

**Figure 4:**
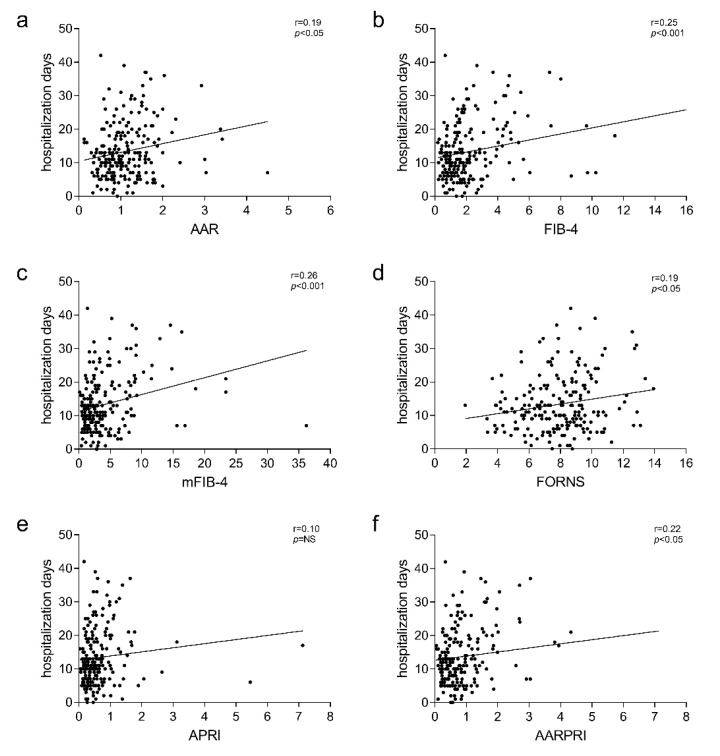
Correlation of AAR (**a**), FIB-4 (**b**), mFIB-4 (**c**), FORNS (**d**), APRI (**e**), AARPRI (**f**) with hospitalization days in discharged patients. The correlation was analysed and estimated using Pearson’s Correlation Coefficient (r). (*p*) indicates statistical significance. Each black dot represents a single patient. Abbreviations: AST to ALT ratio: AAR; fibrosis 4 index: FIB-4; modified FIB-4: mFIB-4; AAR to Platelet ratio: AARPRI.

Furthermore, we found that this association remained when we performed the same analysis only in patients who were discharged, excluding those who succumbed or worsened in condition (Figure 4, *p* < 0.05).

### 3.3. ROC Curve Analysis of Non-Invasive Scores of Liver Fibrosis to Evaluate the Best Predictor for Mortality and Severity in COVID-19

We performed ROC curve analyses of the FIB-4, mFIB-4, FORNS, AARPRI, and AAR scores to define cut-off values and estimate the best prediction for mortality and severity (Table 3).

**Figure 5:**
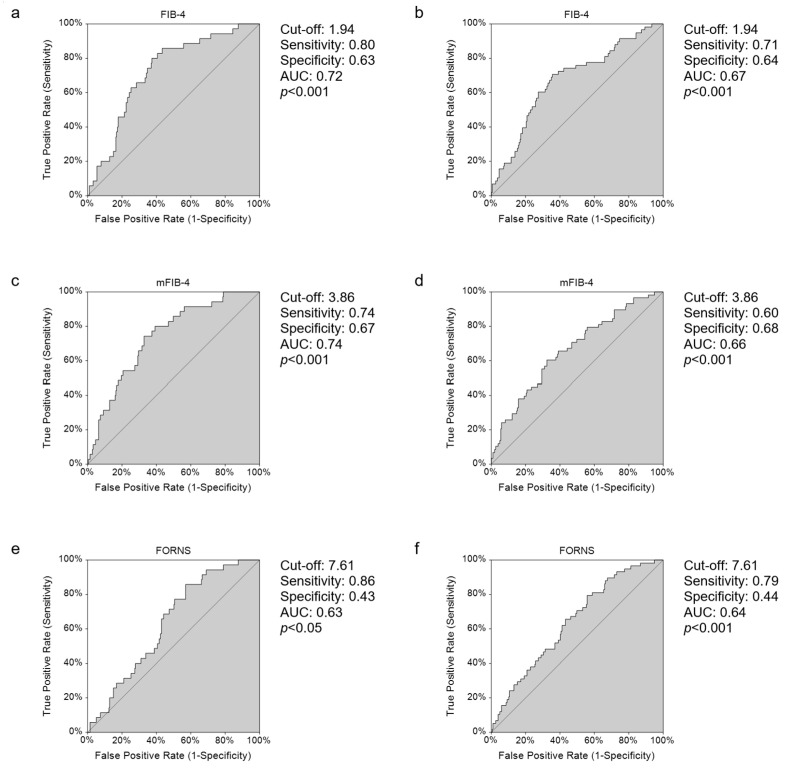
ROC curves for mortality and severity of disease of FIB-4 (**a**,**b**), mFIB-4 (**c**,**d**), and FORNS (**e**,**f**). The graphs indicate cut off values with respective sensitivity and specificity levels and empirical estimation of area under curve (AUC) with upper 1-sided *p*-value (*p*). Abbreviations: fibrosis-4 index, FIB-4; modified FIB-4, mFIB-4.

The empirical ROC curve of FIB-4 was characterised by high specificity and good sensitivity (80% and 63%, respectively) in predicting mortality, with a cut-off value of 1.94 (Figure 5a). The same cut-off value was also identified to predict COVID-19 severity but with a lower sensitivity (Figure 5b). The mFIB-4 score showed a slightly better AUC in predicting mortality, although sensitivity + specificity was lower than FIB-4 (Figure 5c), with the same cut off of 3.86 to predict severity (Figure 5d). The FORNS score also revealed a good sensitivity in predicting disease mortality and severity, while specificity was low (Figure 5e,f). AARPRI showed an AUC of higher than 0.5 but its estimated cut-off values had a very low specificity for mortality and low sensitivity when predicting admission to ICU or death. Conversely, the ROC curves for mortality and severity of AAR presented a low AUC, thereby proving that this index does not discriminate between non survivors patients and those at a higher risk of worse prognosis.

## 4. Discussion

In this study, we demonstrated that non-invasive scores of liver fibrosis predict mortality and clinical outcomes in COVID-19 patients, and also correlate with the length of the hospitalization.

Previous studies found that liver fibrosis was independently associated with mortality, regardless of the demographic characteristics of patients [20]. Specifically, the simple FIB-4 scoring system might predict COVID-19-related mortality, with this connection being likely mediated by SARS-CoV-2-associated damage and monocyte-associated cytokines [16]. Additionally, AAR has already been associated with increased mortality in hospitalised patients [16]. Concerning the FORNS-index, higher values in non survivors were found by Crisan et al. [21]. Similarly to our findings, the APRI score failed to predict COVID-19 mortality. This is probably because it does not consider age, which is instead a proven risk factor for poorer prognosis [22]. On the other hand, in our study AARPRI, which does not consider age, predicts mortality. The increase in these scores may depend on the well-documented increase in liver function tests in COVID-19 patients on admission and is associated with severe disease and increased inflammatory markers, although the pathogenesis for these abnormal values is not fully understood [23]. Consequently, we explored the possibility that these scores may correlate with more severe clinical outcomes. Accordingly, we speculated that the prognosis and severity of the disease may be estimated by the need for oro-tracheal intubation (i.e., in our clinical setting, ICU admission) and the length of hospitalization, which surely increases due to pre-existing comorbidity and COVID-19 related-complications [24]. To the best of our knowledge, only few studies proposed liver fibrosis non-invasive scores, assessed prior to acute COVID-19 illness, to detect the risk of more severe disease [16], increased odds of hospitalization [25], and orotracheal intubation [20]; however, no cut-off values had been estimated.

In fact, in the setting of acute COVID-19 illness, Metabolic Syndrome and its hepatic features, namely NAFLD and NASH that represent an ongoing pro-inflammatory state, might exacerbate the virus-induced cytokine storm, possibly through the hepatic release of pro-inflammatory cytokines, which are responsible for worse prognosis. Moreover, in up to 20% of cirrhotic patients admitted to critical care units, respiratory viruses are usually detected and pneumonia is one of the most common infections in patients with advanced liver fibrosis [26]. A more pronounced baseline systemic inflammation profile in patients with liver fibrosis influences different organs and systems and, with the addition of SARS-CoV-2, the interaction triggers further inflammatory and immune responses, promoting a higher degree of inflammation [27]. Consequently, poorer outcomes in patients with COVID-19 and metabolic disorders might be a result of an “acute on chronic inflammation” process [20]. Furthermore, adipose tissue may serve as a reservoir for SARS-CoV-2 owing to its high level of expression of ACE-2 [28]; this is because, although it is detected in lungs, its expression is lower than in extrapulmonary tissues [2]. For instance, ACE-2 is abundantly expressed within the brush border of enterocytes along the entire intestinal tract [29,30,31]. Unsurprisingly, symptoms such as diarrhea, nausea and/or vomiting, anorexia, and abdominal pain are seen in up to one in five patients with COVID-19 infection [1] and SARS-CoV-2 RNA has also been detected in faeces, even after respiratory symptoms subsided [32]. Additionally, hepatic invasion by SARS-CoV-2 may be possible via constitutively expressed ACE-2, mainly on cholangiocytes and, to a lesser extent, hepatocytes. Thus, it was hypothesised that SARS-CoV-2 induces liver damage primarily in the biliary tract, with the secondary injury and compensatory proliferation of hepatocytes [33]. Microscopically, pathological features of COVID-19 in the liver include moderate macrovesicular steatosis, mild lobular and portal (mainly lymphocytic) infiltration, patchy hepatic necrosis, and both periportal and centrilobular sinusoidal dilatation [34]. This may lead to the elevation of transaminases usually detected in patients with COVID-19 [33], which may also explain why fibrosis non-invasive indexes increase. Consequently, even though plenty of evidence supports the use of non-invasive liver fibrosis scores in predicting mortality, it still remains unclear if liver fibrosis represents a factor affecting prognosis or an early signature of SARS-CoV-2 infection.

In the present study, we did not have the patients’ pre-admission metabolic status and previous anthropometric data. Thus, although we present data for time 0, we cannot exclude the idea that collateral liver damage from virally induced cytotoxic T-cells and the induction of a dysregulated innate immune response could also explain the association between the deranged liver markers and COVID-19 disease severity [35]. Additionally, non-survivors were much older than survivors, and it is well known that older people with SARS-CoV-2 infection may develop severe illness with increased mortality. Nevertheless, one should acknowledge that the analysed fibrotic scores were significantly higher in non-survivors, even without the consideration of age, thus supporting our prediction model. Furthermore, our ROC data revealed that FIB-4 and mFIB-4 scores are the most suitable for predicting mortality and ICU-admission.

Finally, elevated aminotransferases could also originate from myositis rather than liver injury, since myoglobin, hs-Troponin, and LDH values were higher in the non-survivors group. To exclude the influence of this circumstance on the predictive power of non-invasive fibrosis scores, we performed our analysis by considering clinical and biochemical assessment at admission; theoretically, this time period from infection was not sufficient to determine myositis. In addition, no patients reported previous myositis in their anamnesis.

## 5. Conclusions

We found that non-invasive scores of liver fibrosis such as AAR, FIB-4 and mFIB-4, FORNS, and AARPRI strongly predict not only in-hospital mortality but also the length of hospitalization and eventual admission to intensive care units in SARS-CoV-2 infected patients. The strength of the present study is the identification of the cut-off values of FIB-4 and mFIB-4 at admission, which are able to predict disease severity and mortality in COVID-19 patients.

## Figures and Tables

**Table 1:** Non-invasive scores for liver fibrosis with their associated formulas.

Scores	Formulas
AAR	$\frac{AST\;level\left(U/L\right)}{ALT\;level\;\left(U/L\right)}$
FIB-4	$\frac{Age\times AST\;level\left(U/L\right)}{Platelet\;Count\;\left(10^{9}/L\right)\times \sqrt{ALT\;level\;\left(U/L\right)}}$
mFIB-4	$\frac{10\times Age\times AST\;level\left(U/L\right)}{Platelet\;Count\;\left(10^{9}/L\right)\times AST\;level\left(U/L\right)}$
FORNS	7.811−3.131×ln(platelet count)+0.781 ln(GGT)+3.467×ln(age)−0.014(total cholesterol)
APRI	$\frac{AST\;level\left(U/L\right)}{Platelet\;Count\;\left(10^{9}/L\right)\times 100}$
AARPRI	$\frac{AAR}{Platelet\;Count\;\left(10^{9}/L\right)\times 150\;}$

**Table 2:** Baseline characterization of the study population.

Clinical Variable	Survivors	Non Survivors	*p*-Value
*n* (M:F)	233 (130:103)	38 (19:19)	-
Age (years)	67.88 ± 1.027	82.68 ± 1.697	<0.001
Hemoglobin (g/dL)	12.4 ± 0.163	11.9 ± 0.374	NS
WBC (10^3^/µL)	8.46 ± 0.306	9.48 ± 0.798	NS
Monocytes (%)	6.35 ± 0.212	4.39 ± 0.445	<0.001
Lymphocytes (%)	16.4 ± 0.75	9.5 ± 1.25	<0.001
Neutrophils (%)	76.1 ± 0.875	85.3 ± 1.45	<0.001
NLR	7.83 ± 0.539	16.7 ± 2.66	<0.001
Platelet count (10^3^/μL)	249 ± 7.98	217 ± 17.1	NS
Creatinine (mg/dL)	1.51 ± 0.262	1.5 ± 0.213	NS
Urea (mg/dL)	58.3 ± 3.01	97.8 ± 13.2	<0.001
Glucose (mg/dL)	113 ± 3.3	137 ± 12	<0.05
Total Cholesterol (mg/dL)	156 ± 4.06	133 ± 6.1	<0.05
HDL-c (mg/dL)	39 ± 1.48	34.7 ± 2.67	NS
LDL-c (mg/dL)	86.3 ± 3.32	68.6 ± 5.41	<0.05
NON HDL-c (mg/dL)	116 ± 4.31	98.6 ± 5.92	NS
TG (mg/dL)	146 ± 7.54	153 ± 13.1	NS
MHR	0.0148 ± 0.00142	0.0135 ± 0.00202	NS
AST (U/I)	38.8 ± 2.06	37.2 ± 3.32	NS
ALT (U/I)	43.7 ± 3	27.9 ± 2.52	<0.05
GGT (U/I)	68.1 ± 5.35	61.1 ± 14.5	NS
Total Bilirubin (mg/dL)	0.684 ± 0.0502	0.724 ± 0.0896	NS
Ferritin (ng/mL)	709 ± 62.2	712 ± 102	NS
Procalcitonin (ng/mL)	0.469 ± 0.116	3.05 ± 2.3	<0.05
hs-CRP (mg/L)	68.2 ± 7.39	98.4 ± 10.9	NS
ESR (mm/h)	65.4 ± 3.27	61.9 ± 9.56	NS
LDH (mU/mL)	303 ± 9.14	407 ± 29	<0.001
CPK (U/L)	163 ± 32.8	198 ± 59.1	NS
Myoglobin (μg/L)	169 ± 19.4	535 ± 316	<0.05
hs-Troponin (ng/L)	95.9 ± 29.9	574 ± 384	<0.05
NT-proBNP (pg/mL)	3562 ± 932	6449 ± 1531	NS
TSH (mUI/L)	1.84 ± 0.355	1.18 ± 0.362	NS
FT3 (pg/mL)	1.63 ± 0.0559	1.19 ± 0.105	<0.05
FT4 (ng/dL)	1.22 ± 0.0252	1.22 ± 0.0625	NS

Data are presented as mean ± SEM (standard error of the mean). Abbreviations: White Blood Cells: WBC; Neutrophil to Lymphocyte Ratio: NLR; High-density Lipoprotein Cholesterol: HDL-c; Low-density Lipoprotein Cholesterol: LDL-c; Triglycerides: TG; Monocytes to HDL-C Ratio: MHR; Aspartate Transaminase: AST; Alanine Transaminase: ALT; gamma-glutamyl transferase: GGT; high-sensitivity C Reactive Protein: hs-CRP; Erythrocyte Sedimentation Rate: ESR; Lactate dehydrogenase: LDH; Creatine phosphokinase: CPK; high sensitivity Troponin: hs-troponin; N-terminal prohormone of brain natriuretic peptide: NT-proBNP; thyrotropin: TSH; tri-iodothyronine: FT3; thyroxine: FT4.

**Table 3:** ROC curve analyses scores of noninvasive liver fibrosis for FORNS, FIB-4, mFIB-4, AAR, and AARPRI in prediction of mortality and severity.

	CUT-OFF Value	Sensitivity	Specificity	Sensitivity + Specificity	AUC	*p*-Value
FIB-4_MORTALITY	≥1.94	0.80	0.63	1.43	0.72	*p* < 0.001
FIB-4_SEVERITY	≥1.94	0.71	0.64	1.35	0.67	*p* < 0.001
mFIB-4_MORTALITY	≥3.86	0.74	0.67	1.41	0.74	*p* < 0.001
mFIB-4_SEVERITY	≥3.86	0.60	0.68	1.28	0.66	*p* < 0.001
FORNS_MORTALITY	≥7.61	0.86	0.43	1.29	0.63	*p* < 0.05
FORNS_SEVERITY	≥7.61	0.79	0.44	1.23	0.64	*p* < 0.001
AARPRI_MORTALITY	≥0.65	0.82	0.49	1.30	0.69	*p* < 0.001
AARPRI_SEVERITY	≥1.28	0.38	0.82	1.20	0.63	*p* < 0.05
AAR_MORTALITY	≥1.25	0.29	0.81	1.10	0.50	*p* = NS
AAR_SEVERITY	≥0.72	0.59	0.48	1.07	0.48	*p* = NS

Cut off values with respective sensitivity and specificity levels and empirical estimation of area under curve (AUC) with upper 1-sided *p*-value (*p*). Abbreviations: fibrosis-4 index, FIB-4; modified FIB-4, mFIB-4; AAR to Platelet ratio, AARPRI; AST to ALT ratio, AAR.

## Data Availability

The data that support the findings of this study are available from the corresponding author upon reasonable request.
